# How Do Two Types of Exercise Habits Predict Physical Activity with Intention?

**DOI:** 10.3390/bs13110933

**Published:** 2023-11-15

**Authors:** Yoongu Lee, Hyung-IL Lee, Heetae Cho

**Affiliations:** 1 Division of Sports Science, Sun Moon University, Asan 31460, Republic of Korea; ylee0406@sunmoon.ac.kr (Y.L.); soma@sunmoon.ac.kr (H.-I.L.); 2 Department of Sport Science, Sungkyunkwan University, Suwon 16419, Republic of Korea

**Keywords:** physical activity, exercise preparation habit, exercise performance habit, intention, dual-process model

## Abstract

This manuscript investigates how conscious intention and unconscious exercise habits influence physical activity behavior. While prior research has predominantly focused on conscious decision-making, this study applied a dual-process model to explore the impact of intention and habit on physical activity engagement. Out of the 300 questionnaires distributed to students from one university, 282 questionnaires were utilized for data analysis after excluding insincere responses. Intention was measured using a 7-point scale, while exercise habits were assessed using the Self-Report Behavioral Automaticity Index. In addition, physical activity was measured using the Weekly Leisure-Time Exercise Questionnaire. The validity and reliability of measurement tools were confirmed. Data were analyzed using SPSS and AMOS, including correlation analysis, multiple regression, and moderation analysis. Intention, exercise preparation habit, and exercise performance habit were all found to influence physical activity levels significantly. Intention demonstrated the strongest impact, followed by exercise preparation habit and exercise performance habit. This suggests that the research efforts regarding intention conducted before the emergence of the dual process model, which proposes the importance of unconscious thinking patterns, were not in vain. The analysis revealed a statistically significant moderating effect of exercise preparation habit, but not exercise performance habit, in the relationship between intention and physical activity. Exercise preparation habit was identified as a significant moderator, enhancing the relationship between intention and physical activity. This study underscores the importance of considering both conscious intention and unconscious exercise habits in promoting physical activity. The findings challenge the prevailing emphasis on conscious decision-making and highlight the need for a more comprehensive understanding of unconscious behavior in health behavior interventions. This study is expected to arouse academic interest in the often-neglected area of unconscious behavior.

## 1. Introduction

Physical activity is considered essential for leading a healthier life. Optimal levels of physical activity have continuously been developed as health guidelines to support people’s engagement in physical activity. For adults, it is recommended to engage in at least 150 min of moderate-intensity exercise or about 75 min of high-intensity aerobic activity per week [1]. Even if these guidelines are not met, low- or moderate-intensity exercise has been shown to be associated with a decrease in depression [2]. In other words, engaging in any physical activity rather than increasing sedentary behavior time can be seen as beneficial. 

Depression is regarded as a major cause of disease burden worldwide, and great importance is attached to physical activity as an important factor in fighting depression, improving cardiovascular and musculoskeletal health, and preventing cancer [3]. For physical health such as cardiorespiratory health, musculoskeletal health, and cancer prevention, physical vitality may be more important than the area of physical activity (leisure-time physical activity, transportation, occupation, housework, etc.), but for the prevention of mental illness, certain areas of physical activity may be important [4]. Evidence from a meta-analysis suggests that leisure-time and transport-related physical activity has a positive relationship with mental health, but work-related physical activity has a positive relationship with mental illness [5]. Accordingly, in order to expect better mental health benefits from physical activity, leisure-time and transport-related physical activity should be encouraged. Despite these benefits, only about half of the study population adhere to physical activity guidelines [6].

Various theories have significantly contributed to understanding the participation in physical activity from different perspectives. Notable theories such as the health belief model, the theory of reasoned action, and the theory of planned behavior have been applied extensively; however, they suggest limitations in interpretations based on individuals’ conscious thought processes [7]. This is substantiated by the findings that intentions only predict 23% of actual physical activity, and changes in physical activity are limited to 5% [8]. Moreover, despite the intention to engage in physical activity, 36% do not participate, a fact confirmed by a meta-analysis. This phenomenon is referred to as the intention–behavior gap [9]. The dual-process model has been proposed to address this gap, indicating that both conscious and unconscious processes influence behavior [10].

Intention exemplifies a conscious process marked by slow, deliberate, and thoughtful decision-making. In contrast, habits represent the unconscious process characterized by quick, automatic, and impulsive cognitive attributes. Scholars may use varied terms to describe the two types of mind; however, they generally agree on the division between the intuitive mind, which has unconscious, contextualized, rapid, and automatic cognitive attributes, and the reflective mind, which has controlled, slow, decontextualized, analytic, and deliberative attributes [10].

Rhodes and Bruijn [7] suggested the need for additional structures such as self-regulation or automatism, given the limitation that the likelihood of intention developing into behavior is 42%, as identified through a meta-analysis. The research supporting this proposition was initiated by a study that suggested that tasks acquired through easy and repetitive learning are processed quickly and easily through automatic processes, while difficult and less familiar tasks require conscious attention, utilizing more cognitive capacity [11]. A systematic review reported that while the conscious process may explain physical activity, it is directly related to the unconscious process [12]. Accordingly, it was suggested that the unconscious process of habit is interdependent with intention. This claim is backed by a study examining how intention and habit together affect physical activity [13]. It leads to the assumption that when the intention for physical activity is weak since habit significantly correlates with physical activity, a mutually complementary relationship between intention and habit should be leveraged for promoting physical activity. Furthermore, a meta-analysis testing the impact of habit verified that it has an effect size (r = 0.43) similar to that for intention and physical activity [14].

While previous studies have clarified that habits contribute to physical activity, some researchers opine that habits should be classified into two dimensions [15]: habitual instigation and habitual execution. Habitual instigation is defined as the degree of automatic and cognitive response before making a behavioral decision; it refers to the degree of behavior before initiating additional actions (preparing sneakers, moving to the gym, etc.) to participate in physical activity. Habitual execution is defined as the process of all actions towards physical activity; it refers to all actions performed (preparing exercise materials, moving, exercising, etc.) to engage in physical activity. According to research results, habitual instigation better predicts the frequency of behavior and serves as a means to maintain changed behavior, making it habitual. On a different note, preparatory action was identified as a mediator in the relationship between action planning and physical activity, with action planning and preparatory action playing sequential mediating roles between intention and physical activity [16].

Based on these findings, Kaushal, Rhodes, Meldrum, and Spence [17] classified the types of habit into exercise preparation habit and exercise performance habit and tested the causal relationship with physical activity along with intention. They defined exercise preparation habits as actions pertaining to preparing exercise clothes and going to exercise facilities and exercise performance habits as exercise actions performed in the facility. Among intention, exercise preparation habit, and exercise performance habit, only intention and exercise preparation habit were identified as statistically significant predictors of physical activity, thus demonstrating that in explaining physical activity, habits can be classified into two stages.

Rebar et al. [13] pointed out the interaction between intention and habit in influencing physical activity. Their analysis, however, did not differentiate between exercise preparation habit and exercise performance habit and leaves room for improvement as a tool for measuring habit. Given that the exercise preparation habit is a more potent predictor of physical activity than the exercise performance habit, further analysis of their respective interactions with intention is imperative. Hence, this study aims to examine the moderating effects of exercise preparation and performance habits on the relationship between intention and physical activity. The results of this study will elucidate the process of engaging in physical activity and contribute to developing more effective intervention strategies.

Based on the research model, the following hypotheses have been formulated:

**H1.**  *Intention, exercise preparation habit, and exercise performance habit of university students participating in physical education classes will influence physical activity.*

**H2.**  *The stronger the exercise preparation habit of university students participating in physical education classes, the greater the impact of intention on physical activity.*

**H3.**  *The stronger the exercise performance habit of university students participating in physical education classes, the greater the impact of intention on physical activity.*

## 2. Materials and Methods

### 2.1. Participants

According to the results of analyzing physical activity reports from countries around the world, Korean youth’s physical activity participation score is equivalent to grade D-, ranking 37th out of 57 countries, the lowest in the world [18]. The target population of this study was students currently enrolled at A University and attending general physical education classes. In social science research, it is generally considered reliable and acceptable to derive results from approximately 300 questionnaires, and many studies utilize a sample size around this number. Corroborating this, Boomsma [19] argued that results from a path analysis can be deemed trustworthy with a minimum sample size of 200. After explaining this study’s objectives, questionnaires were distributed to students who voluntarily expressed a desire to participate. Data were collected over three months, starting from September 2021, using convenience sampling. Out of the 300 questionnaires distributed, 282 questionnaires were utilized for the analysis after excluding insincere responses. Table 1 outlines the respondents’ demographic characteristics.

### 2.2. Measurement Tools

#### 2.2.1. Intention

Intention was assessed using the questionnaire employed in the study by Caperchione, Duncan, Mummery, Steele, and Schofield [20]. This tool, adapted from the questionnaire proposed as a measurement tool for Ajzen’s [21] Theory of Planned Behavior, consists of two items rated on a 7-point scale. The intention developed on a 7-point scale was converted to a 5-point scale and used. An example of a survey item is “I intend to be physically active for 30 min on most days for the next month”.

#### 2.2.2. Exercise (Preparation/Performance) Habit

To measure the two types of exercise habits, this study employed the Self-Report Behavioral Automaticity Index (SRBAI), developed by Gardner, Abraham, Lally, and de Bruijn [22]. This index includes four items, each rated on a 5-point Likert scale. To differentiate between exercise preparation and performance habits, this study, following the approach of Kaushal et al. [17], adapted the item prompts. To exercise preparation habit items, the phrase “When I prepare for exercise…” was added, and to exercise performance habit items, the phrase “When I exercise…” was added.

#### 2.2.3. Physical Activity

To measure the respondents’ physical activity, the Weekly Leisure-Time Exercise Questionnaire developed by Godin and Shephard [23] was used, with questions adapted by Kim, Cardinal, and Lee [24]. The total amount of physical activity was calculated using the following formula based on the response to each exercise level:

Weekly leisure time activity score = (9 ∗ strenuous) + (5 ∗ moderate) + (3 ∗ mild)

### 2.3. Data Processing

Data analysis was conducted using the SPSS 18.0 and AMOS 18.0 software programs. A frequency analysis was performed to examine the demographic characteristics of the participants. Confirmatory factor and reliability analyses were employed to assess the validity and reliability of the measurement tools. Furthermore, correlation analysis was performed between measurement variables. Multiple regression analysis was performed to evaluate the causal relationship between intention, exercise preparation habit, and exercise performance habit regarding physical activity. Finally, a hierarchical regression analysis was carried out to explore the moderating effects of exercise preparation and performance habits on the relationship between intention and physical activity. In this study, the statistical significance level was set at 0.05.

## 3. Results

### 3.1. Measurement Model

In this study, a questionnaire was created based on previously developed measurement tools, and content validity was secured from two sports psychology professors. In addition, confirmatory factor analysis was conducted to test construct and convergent validity. The results were outlined in Table 2, and the confirmatory factor analysis resulted in the goodness of fit for all factors with χ^2^ = 114.712, df = 30, TLI = 0.917, CFI = 0.944, and RMSEA = 0.100, meeting the criteria presented by Hu and Bentler [25]. The reliability coefficient (Cronbach’s alpha) for intention in this study was 0.951. Additionally, the reliability coefficients for exercise preparation and performance habits were found to be 0.838 and 0.791, respectively. The results of convergent validity testing with the construct reliability (CR) and average variance extracted (AVE) also revealed no problem by exceeding the cutoff [26].

### 3.2. Correlation Analysis

Correlation coefficients were computed to examine the relationships among intention, exercise preparation habit, exercise performance habit, and physical activity, as shown in Table 3, along with the mean and standard deviation for each variable. It was found that physical activity is positively correlated with intention (r = 0.492), exercise preparation habit (r = 0.316), and exercise performance habit (r = 0.129). Additionally, all correlation coefficients between the variable pairs were observed to be 0.80 or below, indicating no issues with multicollinearity.

### 3.3. The Impact of Intention, Exercise Preparation Habit, and Exercise Performance Habit on Physical Activity

The analysis results of the impact of intention, exercise preparation habit, and exercise performance habit on physical activity are detailed in Table 4. The regression model accounted for 33.3% of the variance, yielding an F-value of 46.183 at a significance level of *p* < 0.001. All variables considered in the multiple regression analysis were found to have statistically significant effects, with intention (β = 0.398) found to be most impactful, followed by exercise preparation habit (β = 0.314) and exercise performance habit (β = 0.288).

### 3.4. Moderating Effect of Exercise Preparation Habit on the Relationship between Intention and Physical Activity

**Figure 1:**
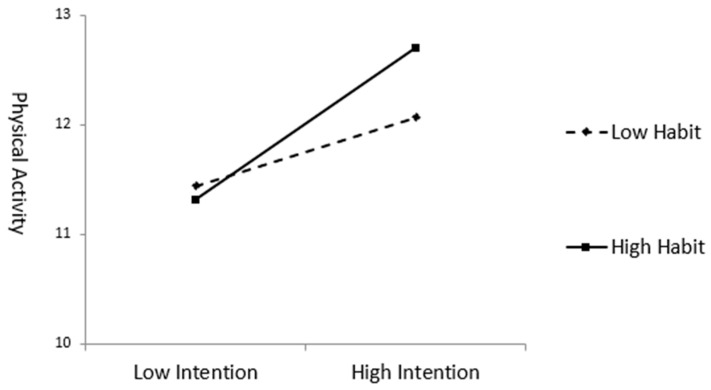
Moderating effect of exercise preparation habit.

Figure 1 and Table 5 present the results of testing the moderating effect of the exercise preparation habit on the impact of intention on physical activity. The test of a moderating effect involves an interaction term, posing a potential risk of multicollinearity between the independent and moderating variables. The interaction term was created to mitigate this risk by subtracting the average value from each intention and exercise preparation habit score and multiplying the mean-centered variables.

In the first stage (Model 1), intention was found to have a significant effect (β = 0.492) on physical activity, explaining 24.2% of the variance. In the second stage, Model 2 was constructed by adding the moderating variable of exercise preparation habit. This addition resulted in a statistically significant effect (β = 0.171), with the explanatory power significantly increasing by 2.6% (*p* < 0.01). In the third stage, Model 3 was constructed by adding the interaction term to test the moderating effect of exercise preparation habit. This addition resulted in a statistically significant effect (β = 0.126), with the explanatory power significantly increasing by 3.2% (*p* < 0.001). Thus, exercise preparation habit was found to moderate the effect of intention on physical activity.

### 3.5. Moderating Effect of Exercise Performance Habit in the Relationship between Intention and Physical Activity

A moderating effect test was conducted to investigate whether the effect of intention on physical activity is affected by exercise performance habit; the results are displayed in Table 6. Moderating effect testing involves an interaction term; this process poses a potential risk of multicollinearity between the independent and moderating variables. To counter this, the interaction term was created by subtracting the average value from each score of intention and exercise performance habit and multiplying the mean-centered variables.

In the first stage (Model 1), intention was found to have a significant effect (β = 0.492) on physical activity, accounting for 24.2% of the variance. In the second stage, exercise performance habit was introduced as a moderating variable in Model 2, yielding a statistically significant effect (β = 0.148) and an increase in explanatory power by 2.2% (*p* < 0.01). In the third stage, Model 3 was constructed by adding the interaction term to Model 2 to test the moderating effect of exercise performance habit. The interaction term had no statistically significant effect. While the explanatory power increased by 0.2%, the difference did not meet statistical significance, indicating that exercise performance habit does not moderate the effect of intention on physical activity.

## 4. Discussion

This study was conducted in response to the need for a differentiated understanding of exercise habits, given the considerable contribution to predicting physical activity. The derived results appear worthy of critical examination.

First, not only intention, which can be considered a conscious process in decision-making but also exercise preparation habit and exercise performance habit, which can be considered unconscious processes, were found to have a positive effect on physical activity. Among these three variables, intention (β = 0.398) had the strongest impact on physical activity. This suggests that the research efforts regarding intention conducted before the emergence of the dual process model, which proposes the importance of unconscious thinking patterns, were not in vain. In a study by Kaushal et al. [17], intention was found to make the largest contribution to physical activity, consistent with this study’s findings. However, their finding that exercise performance habit had no statistically significant effect differs from the results of this study. According to a meta-analysis, intention can explain about 33% of the change in future physical activity [8]. Nevertheless, given that intention alone cannot reliably predict physical activity, it is necessary to consider other factors that can influence behavior alongside intention.

As explored above, while a robust predictive relationship between intention and physical activity is anticipated, exercise habits, classified as unconscious thought processes, are emerging as a supplementary alternative to intention. Exercise habits demonstrate a positive association with physical activity when intention is weak, emphasizing the importance of habits [13]. Consequently, the need was raised to distinguish between habitual instigation and habitual performance and examine them as exercise preparation habit and exercise performance habit, respectively [27].

Based on this, a moderation effect analysis was conducted to explore the impact of exercise preparation and performance habits on the influence of intention on physical activity. The analysis revealed a statistically significant moderating effect of exercise preparation habit, but not exercise performance habit, in the relationship between intention and physical activity. Limited research has been conducted on the differential effects of these two facets of exercise habits. Phillips and Gardner [27] confined the prediction of exercise frequency to exercise preparation habit, suggesting its changes directly impact exercise frequency. Likewise, Kaushal et al. [17] found no significant influence of exercise performance habit on physical activity, further emphasizing the prioritization of preparation habit in behavioral implementation such as physical activity participation. Echoing these findings, Gardner [28] highlighted the importance of preparation habit in predicting simple and complex behaviors, negating the need to segregate the level of behavioral tasks.

“Preparation habit” refers to the automatic performance of behavior triggered by behavioral cues, bypassing a conscious decision-making process, while “performance habit” pertains to the automated stages of sequentially executing the related behavior [29]. This delineation implies the significance of habituating the essential processes in the preparatory stage for enhancing physical activity. Such a strategy is crucial as the robustness of the preparation habit substantially influences the decision to perform a behavior,

Nevertheless, it should not be overlooked that intention is always closely associated with exercise habits. According to a study by Di Maio et al. [30], if the level of physical activity habit is low, high intention can predict physical activity, maintaining a mutually complementary relationship.

The theory validated in this study holds a high practical relevance, particularly for field applications. Despite the recognized benefits of physical activity, the challenge of low exercise participation may find partial resolution through the insights provided by this study. Given that intention does not always translate into action, exploring the interaction between exercise habit and intention can prove valuable. The findings offer practical guidance for on-site experts, such as exercise instructors, presenting strategies for effectively motivating participants into continuous engagement.

By investigating the influence of both conscious and unconscious thoughts and behaviors on specific activities, this study is expected to spark research interest in thus far overlooked unconscious behavior. Additionally, there is a need to explore methodologies for stimulus–response research other than surveys to furnish more substantial evidence in related fields. It is expected that theoretical verification will be carried out from multiple perspectives, as interventional studies related to exercise habits are being conducted based on the accumulated research results, promoting broader participation in physical activity. This study has several limitations. First, it primarily includes women as research participants, with a majority falling within the 19–24 age range. Thus, future research should aim to diversify the participant demographics for a more comprehensive understanding. The second limitation of this study lies in its use of a self-report questionnaire to assess exercise habits, an approach aimed at evaluating the impact of unconscious thoughts, unlike intention. This method raises concerns about the reliability of self-reported data in accurately reflecting unconscious thought processes, underscoring the need for additional validation research to address this issue.

## 5. Conclusions

Most studies with a theoretical approach to boosting physical activity have primarily focused on factors related to an individual’s conscious thinking, making a significant contribution to academia by elucidating causality. Despite the co-occurrence of conscious and unconscious actions, interest in unconscious behavior has remained relatively low. As such, this study applied the dual process model in an attempt to explore individual behavior in its two facets: intention towards behavior, classified as conscious thinking, and habit, classified as unconscious behavior.

The results, according to the hypothesis proposed in this study, were as follows. First, intention, exercise preparation habit, and exercise performance habit were found to have a significant impact on physical activity. Second, exercise preparation habit positively moderated the influence of intention on physical activity. In other words, as the exercise preparation habit becomes stronger, the influence of intention on physical activity increases. Third, the moderating effect of exercise performance habit on the influence of intention on physical activity was not statistically significant. The analysis results of this study suggest a crucial need for reinforcement in the preparatory process area to realize intended behaviors in implementing health behaviors such as physical activity. Accordingly, future research on the development and application of intervention programs that strengthen exercise preparation habits has sufficient value to be used as a physical activity promotion strategy. Additionally, the performative aspect of exercise habits emerges as a supportive force that sustains altered behavior rather than merely enhancing behavioral areas. This study is expected to arouse academic interest in the often-neglected area of unconscious behavior.

## Figures and Tables

**Table 1:** Demographic characteristics.

Variable		n	%
gender	Male	98	34.8
	Female	184	65.2
grade	Freshman	67	23.8
	Sophomore	75	26.6
	Junior	75	26.6
	Senior	65	23.0
age	19–24	250	88.7
	25–29	32	11.3

**Table 2:** Confirmatory factor analysis results.

Factor	Item	Estimate	SE	CR	AVE
Intention	1	0.919	0.383	0.897	0.813
	2	0.994	0.038		
Exercise preparation habit	1	0.703	0.541	0.847	0.582
	2	0.721	0.419		
	3	0.849	0.351		
	4	0.807	0.402		
Exercise performance habit	1	0.514	0.515	0.805	0.524
	2	0.518	0.401		
	3	0.674	0.659		
	4	0.940	0.123		

**Table 3:** Correlation analysis.

	1	2	3	4
Intention	1			
Exercise preparation habit	0.334 **	1		
Exercise performance habit	−0.037	−0.456 **	1	
Physical activity	0.492 **	0.316 **	0.129 *	1
M	3.84	3.05	2.87	28.00
SD	1.10	0.86	0.84	21.85

* *p* < 0.05, ** *p* < 0.01.

**Table 4:** Results of multiple regression analysis.

Independent Variable	B	SE	β	*t*	*F*
(Constant)	−48.298	7.455		−6.478 ***	46.183 ***
Intention	7.927	1.045	0.398	7.582 ***	
Exercise preparation habit	8.014	1.503	0.314	5.333 ***	
Exercise performance habit	7.457	1.441	0.288	5.173 ***	

R = 0.577, R^2^ = 0.333, adjusted R^2^ = 0.325; Durbin–Watson = 2.166; *** *p* < 0.001.

**Table 5:** Moderating effect of exercise preparation habit.

Dependent Variable	Model		B	SE	β	*t*	R^2^	∆R^2^
Physical activity	1	(Constant) Intention	−9.646 9.808	4.135 1.036	0.492	−2.333 * 9.467 ***	0.242	0.242 ***
	2	(Constant) Intention (A) Habit (B)	−5.282 8.671 4.352	4.301 1.082 1.385	0.435 0.171	−1.228 8.012 *** 3.142 **	0.268	0.026 **
	3	(Constant) Intention (A) Habit (B) A × B	−11.879 10.033 3.202 4.373	4.608 1.128 1.396 1.235	0.504 0.126 0.190	−2.578 * 8.893 *** 2.294 * 3.540 ***	0.300	0.032 ***

* *p* < 0.05, ** *p* < 0.01, *** *p* < 0.001.

**Table 6:** Moderating effect of exercise performance habit.

Dependent Variable	Model		B	SE	β	*t*	R^2^	∆R^2^
Physical activity	1	(Constant) Intention	−9.646 9.808	4.135 1.036	0.492	−2.333 * 9.467 ***	0.242	0.242 ***
	2	(Constant) Intention (A) Habit (B)	−10.065 9.917 3.835	4.085 1.023 1.333	0.498 0.148	−2.464 * 9.690 *** 2.878 **	0.264	0.022 **
	3	(Constant) Intention (A) Habit (B) A × B	−9.807 9.858 3.620 0.850	4.104 1.028 1.368 1.197	0.495 0.140 0.038	−2.390 * 9.591 *** 2.647 ** 0.710	0.266	0.002

* *p* < 0.05, ** *p* < 0.01, *** *p* < 0.001.

## Data Availability

The data presented in this study are available on request from the corresponding author. The data are not publicly available due to privacy.
